# On measuring nanoparticle toxicity and clearance with *Paramecium caudatum*

**DOI:** 10.1038/s41598-019-45353-2

**Published:** 2019-06-20

**Authors:** Richard Mayne, Jack Morgan, James G. H. Whiting, Neil Phillips, Andrew Adamatzky

**Affiliations:** 1 Unconventional Computing Laboratory, University of the West of England, Bristol, BS16 1QY UK; 2 Faculty of Health and Applied Sciences, University of the West of England, Bristol, BS16 1QY UK; 3 Health Technology Hub, University of the West of England, Bristol, BS16 1QY UK

**Keywords:** Microscopy, Nanoparticles, Chemotaxis, Data acquisition, Environmental impact

## Abstract

As the extent to which aquatic environments are polluted with nano-scale objects is becoming known, we are presented with an urgent need to study their effects on various forms of life and to clear and/or detoxify them. A range of methods exist to these ends, but a lack of inter-study comparability arising from an absence of experimental standardisation impedes progress. Here we present experiments that demonstrate measurement of orchestrated uptake and clearance of two environmentally-relevant nano- and micromaterials by a model aquatic microoraganism, *Paramecium caudatum*. Experiments were based on a simple, modular, multi-chamber platform that permits standardised control of organism behaviour and measurement of variables relevant to the study of nanotoxicology, including nanomaterial chemotaxis assays, bioaccumulation and deleterious effects on cell motility systems. Uptake of internalised materials may be estimated through the addition of a low-cost fluorescence spectrometer. *P*. *caudatum* cells can clear an estimated 0.7 fg of contaminant materials (or 161 of the particles used) per cell over a 5 mm distance per 6 hour experiment, whilst suffering few short-term adverse effects, suggesting that the organism and the platform used to investigate their properties are well-suited to a range of laboratory and field-based nanotoxicological studies.

## Introduction

Since the advent of the nanotechnology revolution, biological matter is being exposed to nano-scale material, both through deliberate means (e.g. the use of superparamagnetic iron oxide nanoparticles, SPIONs, as contrast agents in MRI technologies^1^), as well as accidental exposure to environmentally-dispersed NMs, which are an emerging class of environmental pollutant^2^. Significant doubt still remains pertaining to the effects of nanomaterials on biological matter, despite increased efforts during the past decade to characterise the phenomenon of nanotoxicology. Inert materials may be rendered reactive, immunogenic or otherwise harmful to life when fabricated in nanoscale quantities^3^; environmental and industrial exposure to nanomaterials, even those touted as being biocompatible (including SPIONs) have been demonstrated to induce states synonymous with disease, including increases in inflammation markers and intracellular reactive oxygen species^4^.

Data are even more sparse regarding the effects of nanomaterials on non-human forms of life, especially at the environmental level, although the data available indicate that nanomaterials may be disproportionately more toxic to lower forms of life and have the potential for bioaccumulation. To continue the example of SPIONs, these have been found to disrupt reproduction in freshwater protists and plants^5^, although the authors’ previous investigations have demonstrated that SPIONs are comparatively non-toxic in a number of model organisms including slime mould^6,7^ and the ciliate *Paramecium caudatum*^8^.

In previous publications, we have argued that manipulation of nano- and micro-particles in ciliated microorganisms such as *P*. *caudatum* constitutes a natural form of ‘sorting’^9–11^, i.e. controlled differential manipulation of environmentally-dispersed objects, based on active discrimination between their physical properties. As *P*. *caudatum* not only preferentially ingests several varieties of nano-scaled material but also concentrates them in intracellular vesicles^12–14^, we find it feasible to suggest that such organisms may be employed to clear and, potentially, partially detoxify (through their assembly into micro-scaled objects) nano-scaled environmental contaminant particles. The immediate application for this is nanotoxicology, as test methodologies in the field tend to lack standardisation^15^, especially where bioaccumulation is concerned. Future applications may include biological intervention for real-world contaminant clearance applications.

**Figure 1:**
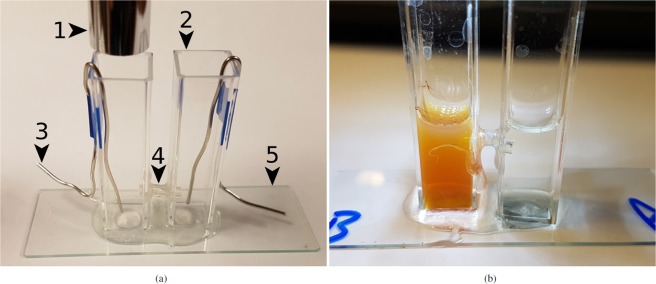
Photographs of the experimental environment. (**a**) Components: (1) Microscope; (2) Cuvette; (3) Pt electrode; (4) Link tube; (5) Base, glass microscope slide. (**b**) Typical chemotaxis experiment. *P*. *caudatum* cells were inoculated into chamber *A* and MNP suspension was introduced into chamber *B* (rusty discolouration). Photo taken at t = 6 h; a sample from *B* revealed cells had migrated $A\to B$ and begun to internalise the MNP suspension (see text for details).

This investigation details a feasibility study into the use of live *P*. *caudatum* cells for nanomaterial clearance (uptake and controlled transport), based on a low-cost, easily fabricated and operated platform that was designed with the aim of standardising the control of microorganisms and measuring key variables relevant to nanotoxicology (Fig. 1). The nanomaterials used, fluorescent latex particles (FLPs) and magnetite nanoparticles (MNPs), were chosen for their being analogous to microplastic particles, an emerging environmental contaminant^16^ and for their increasing use in human medicine^1^, respectively.

Below, we estimate the rate of particle clearance by *P*. *caudatum* and describe the most efficacious methods to control organism migration to and from chambers contaminated with nano- and micromaterials, based on exploitation of well-established mechanisms for influencing their chemo- and galvanotaxes^17,18^. We also present methods to automate output through creating a heterotic computing device based on a low-cost fluorescence spectrometer. We conclude by discussing the open questions and limitations of these techniques.

## Results

### Controlling migration between experimental environment chambers

#### Chemotaxis

All additives — yeast (attractant control), FLPs, MNPs and a 50:50 mixture of FLPs and MNPs — were found to induce migration from chamber $A\to B$ significantly faster than controls with no additives; whilst FLPs were an attractant, their effect was significantly less strong than yeast. Both MNPs and a mixture of MNPs and FLPs were found to be as strong an attractant as yeast (Table 1).

**Table 1:** Comparative strength of chemoattractants represented as the minimum time for at least 1 cell to have migrated between chamber $A\to B$.

	Mean (hrs)	St. Dev.	p*	p**
No additive control	27.5	3.35	—	—
Yeast, attractant control	1.6	0.55	0.0001	—
FLPs	4.4	0.55	0.0001	0.0001
MNPs	1.8	0.84	0.0001	0.6666
FLP & MNPs	1.8	0.45	0.0001	0.5447

p*: unpaired t-test p-value for difference in mean verses no additive control; p**: unpaired t-test p-value for difference in means verses attractant control; n = 5 for each treatment. Full datasets are included in Supplementary S2.

#### Galvanotaxis

**Figure 2:**
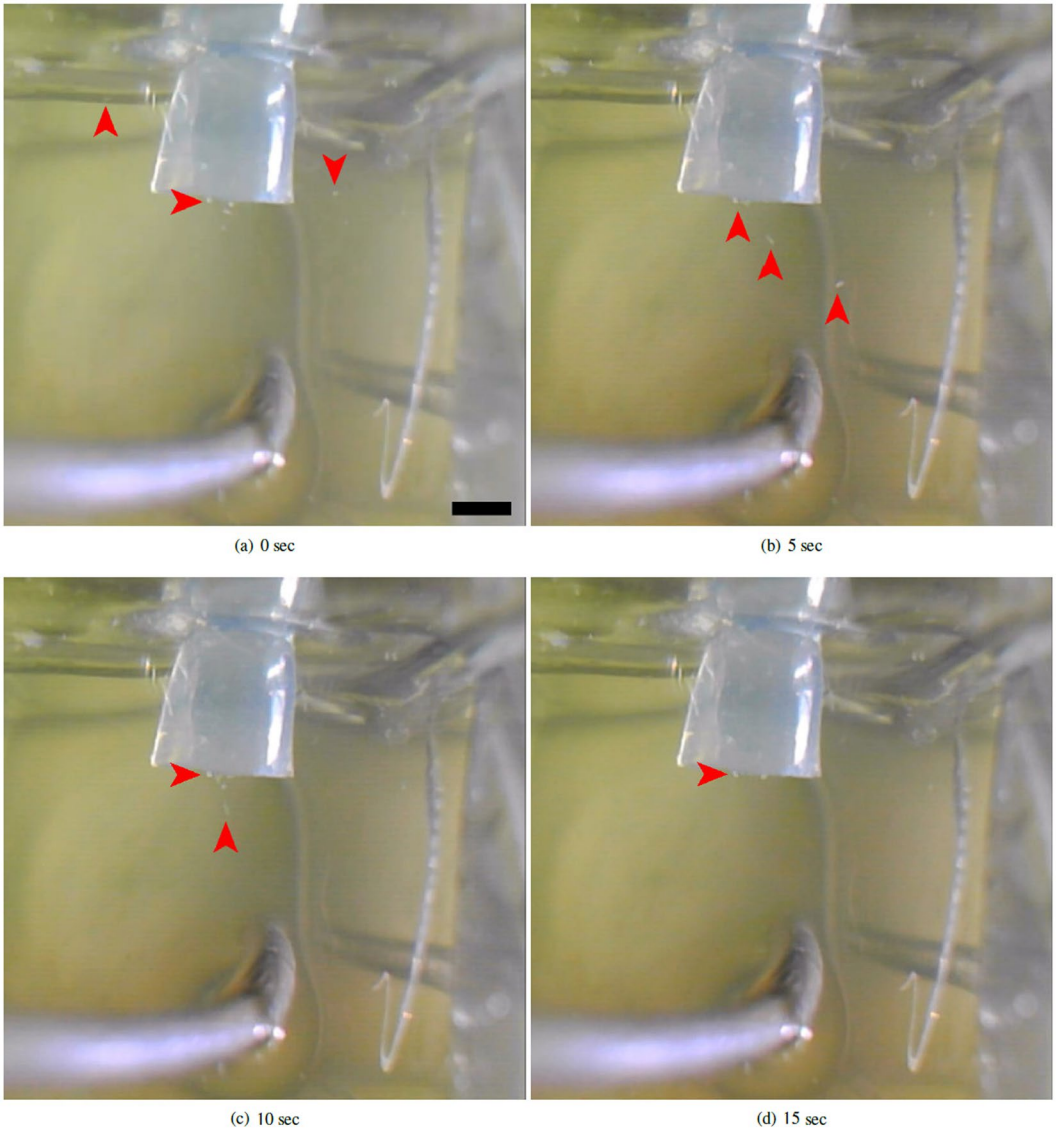
Stereomicrographs to demonstrate the movement of *P*. *caudatum* cells away from a live anode (silver object in bottom central third of images). Several cells (various of which are arrowed) can be seen swimming towards, then down, the linking tube that connects the chambers. Scale bar in [a] 1 mm. Original video included in Supplementary S4.

300 second exposure to the 9 V DC field was found to induce migration to the cathode chamber (*B*) in the vast majority of cells (Table 2, Fig. 2). Cell recovery in *B* was 100% (data not shown).

**Table 2:** Number of cells remaining in anode chamber (*A*) after 300 s exposure to 18 V, 0.3 A DC (repellent) field. (t): test; (c): control; *p*: unpaired t test for difference in mean verses control; n = 10 for each treatment.

	Mean	St. Dev.	*p*
Cells rem. (t)	1.1	1.0	0.0001
Cells rem. (c)	25	0	—

Full datasets are included in Supplementary S2.

### Particle uptake & quantification

**Figure 3:**
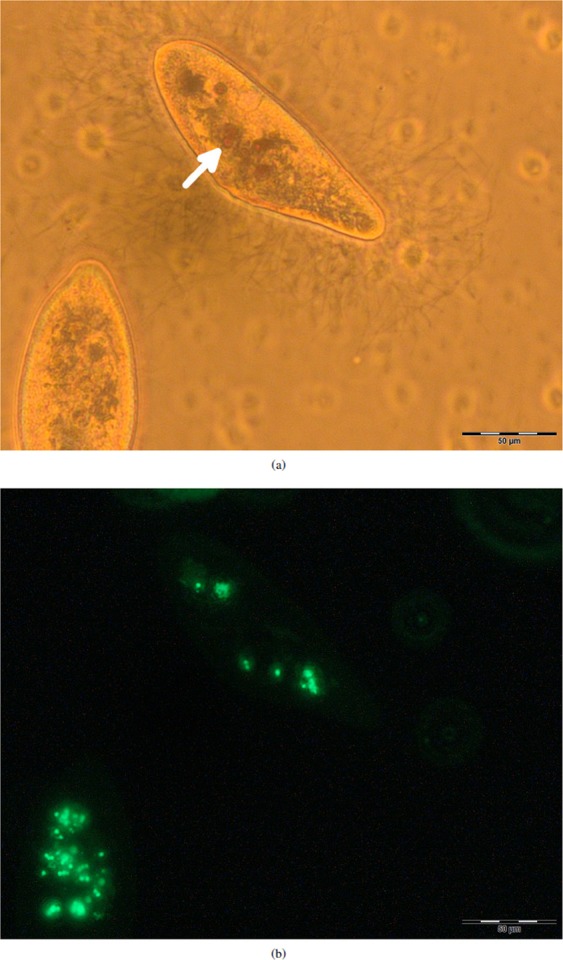
Photomicrographs to show particle intake in fixed *P*. *caudatum* cells exposed to both FLPs and MNPs, showing the same frame with different imaging techniques. (**a**) Brightfield; spherical rust-coloured objects are present in the cell arrowed, corresponding to endocytotic vesicles filled with MNPs. (**b**) Epifluorescence at 488/505 nm, showing FLPs within the cells.

*P*. *caudatum* cells were observed to have internalised both varieties of particle they had been exposed to; cells exposed to FLPs and MNPs simultaneously contained varying quantities of both (Fig. 3), indicating a lack of active discrimination between them during uptake. As cells were fixed immediately after experiments to facilitate imaging, effects of particle application on longevity, morphology etc. were not made.

**Figure 4:**
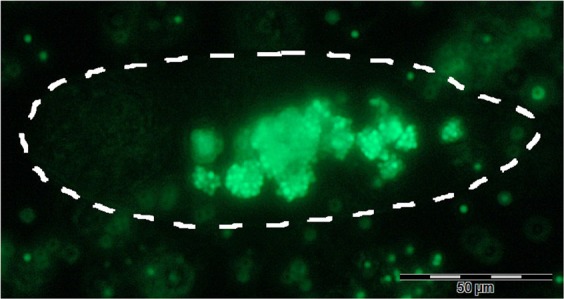
Epifluorescence micrograph used in FLP intake counts, showing a *P*. *caudatum* cell (outlined) post-exposure.

Manual intracellular FLP counts were performed from micrographs (Fig. 4) for use in comparison to spectrometer measurements. Only cells exposed to FLPs in isolation were measured. FLP intake per cell was highly variable with a mean of 161 and st. dev. of 86, range 48–345. The approximate mass of a single latex particle was calculated by the mean volume of a 2 *μ*m latex sphere multiplied by the manufacturer-specified density, is shown in equation 1.

1 $$Mass=Density\times Volume\,1.045\times 4.189\times {10}^{-18}=4.378\times {10}^{-18}\,g$$

We estimate the mass transfer capability for FLPs per *P*. *caudatum* cell, based on the mean and range above, to be 0.70 fg (0.21–1.51 fg). Multiplied to represent the mass transfer by all of the cells in the experiments performed (i.e. 400 cells), this equates to 0.28 pg (0.08–0.60 pg) transferred per 2 ml, per 6 h experiment.

**Figure 5:**
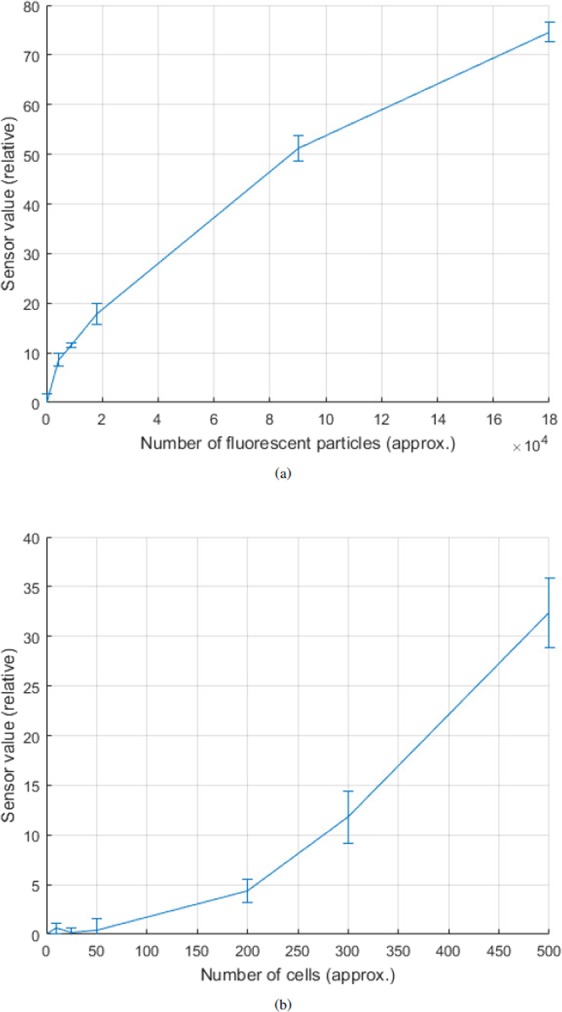
Charts to show spectrometer output. Error bars to 1 S.D. (**a**) Graduated concentration of FLP solution. (**b**) *P*. *caudatum* cells (per ml) exposed to FLPs.

On making calibration measurements, the fluorescence spectrometer device was found to be sensitive to changes in FLP concentration of approximately 4.5 × 10^4^ cell-free FLPs. Increases or decreases in this quantity of FLPs equated to relative sensor changes of 8.33 in a total range of 1024 (st. dev. 1.24) (full dataset available in Supplementary S2). The spectrometer’s calibration curve (Fig. 5a) shows that the sensor’s response was not linear, which was an expected characteristic of the variety of LDR used.

The spectrometer was less sensitive with regards to detecting cells loaded with FLPs (Fig. 5b) but was able to detect a minimum of 200 FLP-treated cells per ml (i.e. approx. 400 organisms total). Accordingly, experiments where chamber $A\to B\to A$ migration was mediated by both chemo- and galvanotaxis, the spectrometer consequently reported a positive identification of the cells with a mean relative sensor value of 4.4 (st. dev. 1.1).

## Discussion

Nanotoxicology research is traditionally conducted by any of a wide variety of methods that may fall under live animal testing, *in vitro* methods (i.e. isolated tissue/cells and cell culture) or acellular systems, which typically constitute studies on colloidal fluids designed to mimic intracellular environments^19^. Animal models are suboptimal test beds due to ethical considerations, prohibitively high costs, long experimental time scales and disparity between the effects of toxic agents on different species. *In vitro* models are also relatively expensive to maintain and do not represent toxicological effects at the organismal level (although, these methods are arguably the most commonly used, with ROS production, cell morphology/growth and organelle morphology/function being the primary determinants of nanotoxicity). None of these methods are conducive to making easily comparable measurements and many require non-standard laboratory equipment or facilities.

Here we presented a simple, cheap (<100 GBP per, assuming that single-use aluminium electrodes are used rather than platinum) device that capitalises on *P*. *caudatum’s* inherent capabilities to migrate under stimuli and uptake objects on the nano-to-micro-scale, towards the study of three principle phenomena, all of which represent active areas of nanotoxicology research:

1. Clearance of environmentally-dispersed nanomaterials by live cells and their intracellular concentration into objects whose size may render them less reactive.
2. Automated detection of cells/cell contents following treatment with nanomaterials through the use of fluorescence, with the possibility of estimating the quantities internalised if the particulates of interest are fluorescent; we demonstrated that it is viable to use FLPs in conjunction with another, non-fluorescent nanomaterial, to represent cases where it is not viable to modify the nanomaterial of interest.
3. Investigation of cell dynamics following treatment with nanomaterials, through studying migration *in situ* and retrieving the cells in comparatively non-contaminated media post-experiment, e.g. to study particle retention and bioaccumulation.

Further, the device, which can be built, operated and modified with minimal expertise, permits rapid and reproducible measurements and may also be used for field work. There is significant scope for adaptation and development of the device, e.g. optimising the spectrometer sampling area, addition of additional chambers. Several limitations in its design may also be addressed by further development, such as the need for a chemoattractant to be present for migratory studies and the requirement for fluorescent materials for digital estimation of particle transfer.

The inclusion of a microprocessor, rather than simple circuitry, also allows for the user to program more complex operations, such as switching of electronically-controlled stimuli, recording data from multiple sensors/experiments concurrently, remotely storing data and real-time analysis, e.g. self-calibration. Whilst ‘DIY’ spectrophotometers are commonplace amongst hobbyists and there exist scientific publications recommending routes towards building devices of varying complexity and cost for educational and ‘citizen scientist’ applications^20,21^, the devices presented here are, to our knowledge, the first fully-modular platform for fluorescence spectrometry adapted for nanotoxicological applications. The designs presented here prioritised balancing cost with ease of assembly and result accuracy, whereas hobbyist prototypes are typically crude (e.g. utilising pinholes to split light, rather than optical filters^22^) and more bespoke, academic prototypes tend to be more sensitive, but correspondingly more expensive and designed for a single, fixed-use^21^.

We offer no data to suggest whether these techniques are scalable, although large-scale environmental clean-up or laboratory waste detoxification are attractive — albeit optimistic — prospects. For completeness, estimated total clearance of FLPs at normal stock culture concentrations of *P*. *caudatum*, ca. 1000 cells per ml (i.e. 2000 cells per experiment) would, using our calculated mean and range values, equate to 1.40 pg (0.42–3.02 pg), or approximately 3.2e5 (1.0e5–6.9e5) individual FLPs transferred per experiment (6 hours). To contextualise our data with estimations of the abundance of micro- and nanoplastic particles in freshwater environments, various tributaries to an urban river in United Kingdom were recently reported to contain an average of 165 particles per kilo, when extracted from silt samples^23^.

The variability in the number of FLPs ingested per *P*. *caudatum* cell indicates that there is likely no set volume or mass of particles that they will attempt to ingest, hence it is unlikely that an estimation of the quantity of MNPs or indeed any other variety of nanomaterial from the data presented here are likely to be of value. The principle difference in the manner in which *P*. *caudatum* cells were observed to respond to the two varieties of contaminant particulate used was their relative speed of migration between chambers: whilst it isn’t surprising that the starch-coated MNPs appeared to act as a chemoattractant, this observation highlights the suitability of the experimental environment for making quantitative measurements on organism–nanoparticle interactions and hence suggests routes towards further research. Our future work will therefore concentrate on evaluating similar devices with further species of motile aquatic microorganism and nanoparticles of interest.

## Methods

### Culture

*P*. *caudatum* were cultivated in a modified Chalkley’s medium enriched with 10 g of dried alfalfa and 40 grains of wheat per litre at room temperature in non-sterile conditions. Cultures were exposed to a day and night cycle but were kept out of direct sunlight. Organisms were harvested in log growth phase by centrifugation at 400 × G prior to being rinsed and resuspended in fresh media.

### Experimental environment

In light of the lack of standardisation of nanotoxicological experiments, the experimental environment (Fig. 1) used was designed on the basis of being a simple, modular platform for making reproducible measurements between a range of organisms and across a range of laboratories. It consisted of two polyethylene fluorimeter cuvettes (named ‘*A*’ and ‘*B*’) measuring 12 × 12 × 44 mm, affixed to the base of a glass microscope slide (75 × 25 × 1.0 mm) with epoxy resin (Araldite, Huntsman, USA). The cuvettes were linked at a point 15 mm above their base by flexible polytetrafluoroethylene (PTFE) tubing, OD 3.0 mm ID 1.0 mm length 5 mm, which were affixed to the cuvettes with epoxy. The connecting tube’s length was sufficient to make the rate of diffusion between the chambers low to negate the possibility of significant quantities of nanoparticles from diffusing between them in the time allotted for each experiment (see Supplementary S1). Prior to each experiment, both chambers were filled with 2.0 ml of sterile culture medium. Care was taken to ensure that fluid levels were equal in both cuvettes and that the linking tube did not become air-locked. Whenever quantities of fluid containing cells or particles were added to a chamber, an equal quantity of fluid was added to the other chamber simultaneously in order to prevent fluid transfer resulting from pressure changes.

A range of initial control experiments demonstrating the suitability of the experimental environments used — showing that *P*. *caudatum* cells do not transfer between chambers for at least 24 hours post inoculation if no stimuli were applied and that no particulate transfer was observed over 24 hours in the absence of cells — are included in Supplementary S1.

### Nano-/microparticle varieties and visualisation

The following two varieties of particulate were chosen for use in all experiments as our previous work has demonstrated that *P*. *caudatum* will favourably ingest both whilst suffering minimal deleterious health effects in the short term (for a characterisation of these nanomaterials and their interactions, please see refs ^8,9^).

- 2.0 *μ*m diameter carboxylate-modified latex microspheres labelled with fluorescein (Sigma Aldrich, Germany) (hereafter, FLPs, ‘fluorescent latex particles’). 100 *μ*l of stock solution (2.5% solids, approx. 7.20×10^9^ particles per ml) was diluted to give a total concentration in 2 ml of fluid of 0.125% solids w/v (approx. 7.20×10^8^ particles).
- 200 nm diameter multi-core magnetite (iron II/III oxide) nanoparticles, prepared with a hydrodynamic starch coating (Chemicell, Germany) (hereafter, MNPs, ‘magnetite nanoparticles’). 100 *μ*l of stock solution (25 mg/ml) was used to give a total concentration for chamber A of 1.25 mg/ml (approximately 2.75×10^11^ particles). Samples were briefly sonicated prior to use.

All observations were made with a Zeiss Axiovert 200 M inverted microscope and data were captured with an Olympus SC50 camera via CellSens software. FLPs were observed through excitation with a 488 nm LED lamp (CoolLed, UK) and MNPs were observed with phase contrast optics. Although the particle size of the MNPs was beyond the resolution of the light microscope, fluid discolouration was quantified through software and a magnet was sometimes employed to draw aggregated MNPs into the field of view; MNPs within *P*. *caudatum* cells were aggregated within endocytotic vesicles in quantities visible under ×400 magnification.

All cells were observed by transferring them to a cavity slide containing 100 *μ*l of fixative, 2% paraformaldehyde in pH 7.2 phosphate buffered saline.

### Controlling migration

Two methods for controlling migration of *P*. *caudatum* cells, one attractant and one repellent, were employed. Attraction was mediated chemically: this decision was made in order to enhance uptake via the organisms’ feeding system and to disrupt normal cellular processes as little as possible. Chemoattractive potential of the two varieties of particulate, both in isolation and in conjunction, were evaluated verses a known chemoattractant, heat-fixed yeast (*Saccharomyces cerevisiae*) suspensions (see Supplementary S1 for culture details).

Approximately 25 *P*. *caudatum* cells in 200 *μ*l of medium were placed in chamber *A* whilst *B* was simultaneously filled with the same quantity of attractant/particle solution (dechlorinated tap water for controls) and the experimental environment was placed onto a stereomicroscope stage, focused on *B*. The chambers were checked every 2–4 hours until a cell was identified, at which the time elapsed since the start of the experiment was recorded as the ‘minimum migration time’.

Repulsion was mediated electrically via two 25 × 1° mm platinum wire electrodes, inserted one per chamber into the experimental environment, based on the well-characterised principle of *P*. *caudatum* galvanotaxis (migration towards the cathode in a DC electrical field). Electrical control of migration was chosen for its rapidity and ability for being dynamically altered.

Similarly to the aforementioned chemotaxis experiments, 25 cells in 200 *μ*l of medium were transferred to chamber *A* whilst *B* was simultaneously filled with an equal quantity of fresh media. The electrodes were connected to a bench-top power supply in a configuration such that the cathode was located in chamber *B*. Initial experimentation demonstrated that 18 V at 0.3 A was sufficient to induce rapid responses from the cells without causing lysis through the duration of the experiment. The experimental environment was placed on the stage of a stereomicroscope focused on chamber *A*. The power supply was switched on for 300 seconds, after which the number of cells remaining in *A* was recorded.

### Measurement of particle uptake

#### Manual counting

Cells were added to chamber *A* at a concentration of approx. 200 per ml (i.e. approx. 400 total) whilst the same quantity of FLP solution was simultaneously added to *B*. The experimental environment was then placed in an opaque box for 6 hours, after which cells were removed from *B* for observation. Counts of FLP intake per cell were made manually. No attempts were made to quantify MNP uptake in this study.

#### Spectrometry

A low-cost fluorescence spectrometer was developed towards creating a simple method for detecting the completion of a nanoparticle clearance operation in organisms exposed to a mixture of a nanoparticle of interest and FLPs. The device was designed to articulate onto a single fluorimeter cuvette and was produced as follows (see Supplementary S2 for parts list).

The light source was a single surface-mount 485 nm light emitting diode (LED) that produced 479 Lux when driven by a benchtop power supply at 3.1 V and 250 mA. The LED was mounted to a custom board with a large aluminium heat-spreader, onto which a 15° collimator lens was affixed in order to focus the light produced. The power supply was switched via a relay controlled by an microprocessor board based on the Arduino/ATmega328 platform (microprocessor code is included in Supplementary S3).

**Figure 6:**
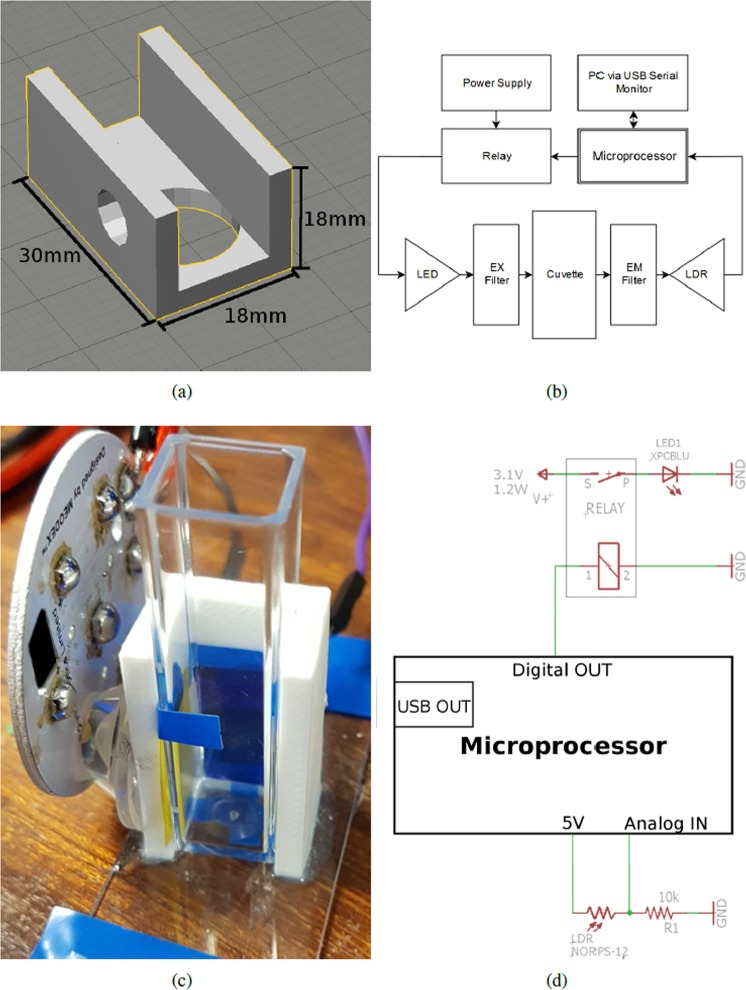
Fluorescence spectrometer. (**a**) 3D printed cuvette adaptor. (**b**) System diagram. (**c**) Photograph of assembled LED with heatsink, LDR (rear), collimator, adapter and cuvette. (**d**) Wiring diagram.

The LED, board and lens assembly was mounted to an experimental environment’s cuvette via a slip-on 3D printed adapter (Fig. 6a), which was generated in OpenSCAD on an open source template^24^. The adapter, which fit snugly around three sides of the cuvette, also served to mount two filters and a light dependent resistor (LDR) at a 90° angle to the light path. The filters used were an excitation bandpass filter (i.e. between the light source and sample) and an emission notch filter (between sample and detector); inspired by ref.^25^, we used low-cost photography lighting gels as filters (Rosco, USA).

The light intensity was measured by the LDR, using a voltage divider with a resistor (10 kΩ), with the output of the voltage divider read by an analogue pin on the microprocessor. The on-board 10-bit ADC converted the analogue signal into a digital value which was sent to the PC via serial monitor USB link. The system is illustrated in Fig. 6; (b) system schematic, (c) assembled and (d) wiring diagram. A photograph of a deconstructed system is included along with a detailed description of its assembly in Supplementary S1.

The operation cycle of the spectrometer was as follows: the LED is switched on for 1 second and the LDR value is read and printed on-screen. The LED then switches off for 1 second and the LDR value is printed again. The first value (*x*) returned gives the sensor value, indicating the quantity of fluorescent compound in the sample, and the second value (*y*) indicates a reference value as an internal control for each reading; therefore, *x*–*y* gives a relative sensor value, which may in turn be converted to a particle/cell count estimation with reference to a calibration curve.

The spectrometer was operated inside an opaque box in order to eliminate interference by ambient light. Initial device evaluation was performed on graduated series of FLPs. A calibration curve was made by adding known quantities of FLPs to 2 ml of fresh media in a fluorimeter cuvette and making measurements. Further calibration measurements were made by adding known numbers of cells that had been exposed to FLPs for 6 hours as per the previous ‘manual counting’ experiments; for small numbers of cells, individual organisms were pipetted in, whereas for larger numbers (>200), centrifugation and dilution in conjunction with manual cell counts were used to achieve approximate concentrations.

Further experiments were performed with both electrodes *in situ* and the spectrometer affixed onto and the cathode immersed in chamber *A*. Approx. 400 cells were added to *A* and FLPs were added to *B*. The cells were allowed 6 hours to migrate to chamber *B* and consume FLPs, after which the electrodes were connected to a power supply for 300 sec. Spectrometer readings were then made.

## Supplementary information

Supplementary Information 1

S4 video

Related Manuscript File

S2 Spreadsheet
