# Supplementary material for: On measuring nanoparticle toxicity and clearance with Paramecium caudatum
Source: Sci Rep. 2019 Jun 20;9:8957. doi: 10.1038/s41598-019-45353-2 (PMC6586663; doi:10.1038/s41598-019-45353-2)
Supplement: Supplementary file 1 — Supplementary Information 1 [file 41598_2019_45353_MOESM1_ESM.pdf]

On measuring nanoparticle toxicity and clearance with *Paramecium caudatum* Supplementary Information

Richard Mayne, Jack Morgan, James G. H. Whiting,
Neil Phillips & Andrew Adamatzky

Key to supplementary information files

1. Text document with various additional experimental details.
2. Spreadsheet for supplementary datasets for various experiments.
3. Arduino sketch (code) for spectrometer device.
4. Video showing *P. caudatum* cell galvanotaxis.

Estimation of diffusion rate between EE chambers

Simple diffusion between both chambers was estimated to take a minimum time of approximately 3 hours 30 minutes: by Equation 1

$$L^2 = 2DT \tag{1}$$

Where L is length, D is the coefficient of diffusion (estimated at maximal rate of 10^{-9} in liquids) and T is time, thus the time to diffuse 5 mm would be 12,500 seconds (208 minutes).

Suitability of experimental environment experiments

Two control experiments were used to determine the suitability of the experimental environments (data are included in Supplementary Information 1). These were:

1. Rate of *P. caudatum* migration between chambers when no external stimuli were applied.
2. Rate of transfer of particulates between chambers in the absence of *P. caudatum* cells.

For (1), approximately 25 cells were transferred to chamber A in 100 μl of fresh culture medium. The experimental environments were placed on the stage of a stereomicroscope which was focused on chamber B. The sample was kept static for the duration of the experiment and was observed regularly every 2–4 hours, until cells were identified. The microscope's halogen lamp was switched off in between observations and the entire setup was exposed to a day/night cycle whilst being kept out of direct sunlight. The mean time before cells were identified was 27.2 hours (st. dev. 3.35 hours, minimum 24 hours).

For (2), both particle varieties (FLP and MNP) were added to chamber A at the start of each experiment. Samples were taken every 4–6 hours by simultaneously drawing 10 μl of fluid from the bottom of both chambers and examining the sample from chamber B. No FLPs or MNPs were observed to have diffused to chamber B over the 48 hour experiments.

Yeast culture and fixation

Saccharomyces cerevisiae were cultured from approximately 20 grains (0.1 g) of freeze dried, commercially-available bread yeast (Allinson, UK) in 10 ml of dechlorinated tap water containing 0.1 g of glucose in an incubator at 22°C, for 1 hour. The cultures were then transferred into boiling tubes and placed in a

100°C water bath for 15 minutes. A few drops of 40% Congo red dye dissolved in ethanol were added (as a stain) before cultures were reserved in a refrigerator until use.

Electronics assembly

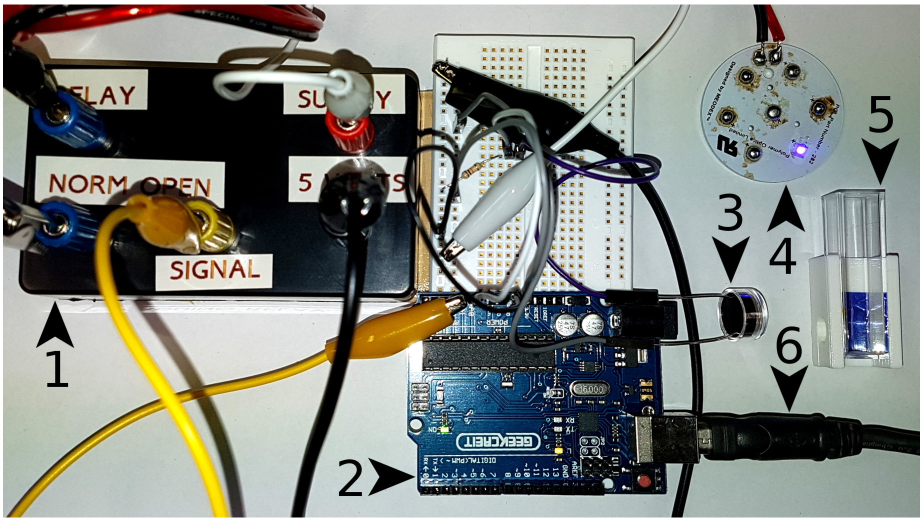

Figure 1: Photograph of a partially-assembled spectrophotometer system. See text for description

1. **Relay**. This component is a standard 5V 5-terminal automotive (Bosch-style) relay, which has been placed in a large project box for illustrative purposes, but may be mounted in a significantly smaller package on the breadboard. The two terminals on the left with blue caps are the 'normally open' and 'normally closed' terminals, which are connected to the LED (component 4). The central, yellow terminal is the 'COM' signal, which is connected directly to the digital out pin on the microprocessor (component 2). The two terminals on the right are connected to a power supply, which in this image is coming from the 5V rail of the microprocessor via the breadboard (again, for illustrative purposes), but in experiments was driven by a 5V bench-top power supply.

2. **Microprocessor & Breadboard**. The microprocessor has two main purposes in this setup: firstly, to provide a signal to the relay via a digital out pin and secondly, to record the output from the LDR (component 3). The breadboard is here used to provide a common power rail and allow for a voltage divider circuit to be made through the use of a 1kOhm resistor. The setup and choice of components for the breadboard are arbitrary and may be adapted to the user's preference. For pin locations and wiring diagram, please see Fig. 6 in the main document.

3. **Light dependent resistor (LDR)**. This component is attached via jumper cables across the 5V rail on the microprocessor and an analog

in pin, via a voltage divider (see Fig. 6 in the main document for wiring diagram). The LDR slots into the 3D-printed cuvette adapter (component 5).

4. **Light-emitting diode (LED) board**. This ultra-bright LED is soldered onto a proprietary surface-mount board. The board has the option to mount multiple LEDs, but as this application only requires one, the remaining surface-mount slots were soldered into an open circuit. Collimator not shown.

5. **Cuvette and adapter**. The cuvettes used are conventional fluorimetry cuvettes. Two filters are stuck to two adjacent external walls. The adapter, which slots onto the cuvette and holds both LDR and LED components, was printed with an Ultimaker 2 3D printer.

6. **Computer connection**. In its current form, the device requires an computer connection via USB.
