# Supplementary material for: On measuring nanoparticle toxicity and clearance with Paramecium caudatum
Source: Sci Rep. 2019 Jun 20;9:8957. doi: 10.1038/s41598-019-45353-2 (PMC6586663; doi:10.1038/s41598-019-45353-2)
Supplement: Supplementary file 3 — Related Manuscript File [file 41598_2019_45353_MOESM3_ESM.docx]

/*

Modified Arduino example code from the publbic domain:

http://www.arduino.cc/en/Tutorial/AnalogInOutSerial

Modification for driving DIY spectrometer in support of paper:

DOI: XXXXXXXXXXXXXXXXXXX

"Programmable ciliature-mediated smart actuating device for micro-object manipulation and delivery, operated by Paramecium caudatum", R. Mayne et al.

*/

const int analogInPin = A0; // Analog input pin that the potentiometer is attached to

const int analogOutPin = 9; // Analog output pin that the LED is attached to

int sensorValue = 0; // value read from the pot

int outputValue = 0; // value output to the PWM (analog out)

void setup() {

// initialize serial communications at 9600 bps:

Serial.begin(9600);

pinMode(A2, OUTPUT);

}

void loop() {

digitalWrite(A2, HIGH); // turn the LED on (HIGH is the voltage level)

// read the analog in value:

sensorValue = analogRead(analogInPin);

// map it to the range of the analog out:

outputValue = map(sensorValue, 0, 1023, 0, 255);

// change the analog out value:

analogWrite(analogOutPin, outputValue);

// print the results to the Serial Monitor:

Serial.print("sensor = ");

Serial.print(sensorValue);

Serial.print("\t output = ");

Serial.println(outputValue);

delay(1000); // wait for a second

// Read with LED off for comparison of dark and light readings

digitalWrite(A2, LOW); // turn the LED off by making the voltage LOW

sensorValue = analogRead(analogInPin);

// map it to the range of the analog out:

outputValue = map(sensorValue, 0, 1023, 0, 255);

// change the analog out value:

analogWrite(analogOutPin, outputValue);

// print the results to the Serial Monitor:

Serial.print("sensor = ");

Serial.print(sensorValue);

Serial.print("\t output = ");

Serial.println(outputValue);

delay(1000);

}
